# Seeing the light brings more food in the deep sea

**DOI:** 10.15252/embj.2023114091

**Published:** 2023-04-13

**Authors:** Ute Römling, Andreas Möglich

**Affiliations:** ^1^ Department of Microbiology, Tumor and Cell Biology Karolinska Institutet Stockholm Sweden; ^2^ Department of Biochemistry Universität Bayreuth Bayreuth Germany

**Keywords:** Metabolism

## Abstract

Cyclic di‐GMP signaling regulates sessile‐to‐motile lifestyle transition and associated physiological and metabolic features in bacteria. The presence of potential cyclic di‐GMP turnover proteins in deepest branching bacteria indicates that cyclic di‐GMP is an ancient signaling molecule. In this issue of The EMBO Journal, Cai *et al* (2023) describe light‐induced activation of a thiosulfate oxidation pathway in the deep‐sea cold‐seep bacterium *Qipengyuania flava*, thus coupling cyclic di‐GMP with the regulation of the global abiotic sulfur cycle.

## Graphical abstract

Recent study links light‐dependent cyclic di‐GMP signaling in a deep‐sea cold‐seep bacterium to the marine sulfur cycle.

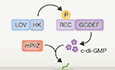

Bioinformatic analyses pinpoint cyclic di‐GMP as the most abundant second messenger in bacteria. Ubiquitously regulating the lifestyle transition between sessility and motility, this signaling molecule has been mainly investigated in animal and plant pathogens, where it not only regulates the trade‐off between environmental survival versus acute virulence but also directs the intracellular lifestyle of microbes (Römling *et al*, 2013). Although the same cyclic di‐GMP‐regulated pathways, such as the biosynthesis of the exopolysaccharide cellulose, are relevant in different environments (Pontes *et al*, 2015), the full breadth of physiological and metabolic regulation by cyclic di‐GMP signaling, as well as most of the ancient associated pathways have not yet been defined.

Among the fundamental pathways controlled by cyclic di‐GMP signaling are the cell cycle, respiration, photosynthesis, cell morphology, and DNA repair. Regulation is exerted at the levels of transcription, translation, and post‐translational modifications. Cyclic di‐GMP turnover proteins, histidine kinases of two‐component systems, and other signaling proteins are inherently modular and can receive input from a multitude of signals, including light and temperature by sensory domains, thus enabling tailored and highly specific responses.

The ocean is a major source of microbial activity and contributes substantially to the global carbon, nitrogen, and sulfur cycles. Oceanic sediments are among the major microbial habitats on Earth. Particular hotspots of microbial activity include hydrothermal vents and cold seeps, which provide a rich source of molecules to promote biogeochemical activities.

Sulfur, the fifth most abundant element on Earth, is essential for all organisms as a constituent of abiotic Fe‐S clusters and organosulfur molecules such as amino acids and prosthetic groups. With an oxidation state ranging from −2 to +6, sulfur is particularly amenable to oxidation and reduction processes. Given the availability of sulfur in form of minerals and gaseous H_2_S, microbial life harnessed the conversion of the sulfur redox state for energy gain and growth. Abiotic sulfur reduction, aerobic and anaerobic oxidation, and disproportionation recur throughout the bacterial domain.

Thiosulfate, both product and substrate of sulfur oxidation in microorganisms, is a central intermediate in sulfur metabolism and the global sulfur cycle. While its oxidation to sulfate was previously considered to be performed via the classical Sox oxidation pathway, a novel two‐step oxidation pathway conducted by the thiosulfate dehydrogenase TsdA, the thiosulfohydrolase SoxB, and subsequently the sulfur dioxygenases SodA/SodB had recently been identified in the alpha‐proteobacterium *Q. flava*, previously designated *Erythrobacter flavus*, isolated from the sediment of a deep sea cold seep (Fig 1; Zhang *et al*, 2020). Widespread among bacteria, this pathway leads to the generation of zero‐valence sulfur via a tetrathionate intermediate. The zero‐valent sulfur is detoxified by transport into or out of the cell and promotes growth *in situ*, as it can be consumed in the next round of oxidation to sulfite (Cai et al, 2022).

**Figure 1:**
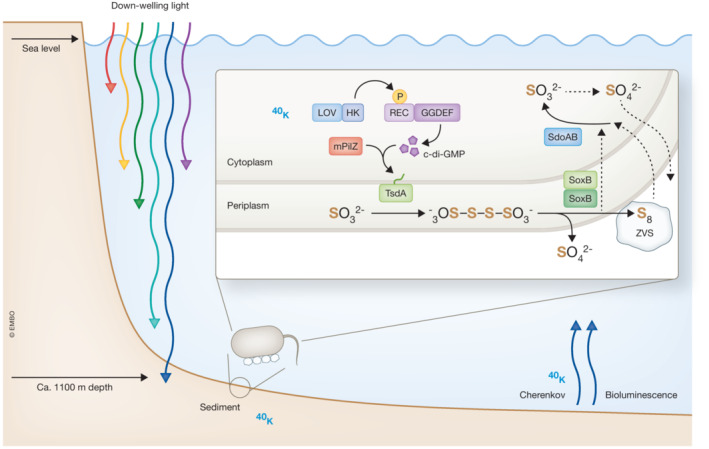
Light‐ and cyclic di‐GMP‐dependent regulation of sulfur metabolism in *Qipengyuania flava* isolated from cold‐seep sediment in the deep sea as investigated in this work A light‐oxygen‐voltage (LOV) histidine kinase is activated by blue light that might originate from sunlight, bioluminescence, or Cherenkov radiation from potassium decay. In turn, the kinase phosphorylates and thereby activates a response regulator (REC) diguanylate cyclase (GGDEF) to produce the widespread second messenger cyclic di‐GMP. Upon cyclic di‐GMP binding, a mPilZ receptor stimulates the enzymatic activity of the thiosulfate dehydrogenase TsdA. In concert with the two SoxB thiosulfohydrolases, TsdA mediates the conversion of thiosulfate (S_2_O_3_^2−^) to elemental, zero‐valence sulfur (ZVS) and SO_4_^2−^ via a tetrathionate intermediate (^−^O_3_S‐S‐S‐SO_3_^−^). The ZVS is deposited as granules, from which it can later be metabolized by sulfur dioxygenases (SdoA/SdoB) via oxidation to sulfite (SO_3_^2−^), which is subsequently spontaneously oxidized to SO_4_^2−^. Note that not all pathway intermediates have been displayed. Dashed line arrows summarize undefined pathways.

Like other organisms, bacteria possess sensory photoreceptors to derive spatial and temporal cues from environmental light. Given its superior penetration through the water column, blue light dominates photoreception in the ocean. Piqued by the observation that *Q. flava* produces significantly more zero‐valent sulfur in light than in darkness when provided with thiosulfate, Cai *et al* presently investigated the molecular basics of light‐dependent activation (Fig 1). Exclusion experiments indicated one of the two histidine kinases with a light‐oxygen‐voltage (LOV) photosensory domain, denoted LOV‐1447, to underlie this phenotype. Originally discovered as the blue light‐sensitive pigments in plant phototropins (Christie *et al*, 1998), LOV receptors abound in the microbial realm, where they mediate light‐dependent adaptations of lifestyle, differentiation, stress responses, and virulence besides other processes. Among seven proteins identified in the *Q. flava* genome that comprise receiver domains (REC), four interacted with LOV‐1477. Being the sole response regulator with a covalently connected output domain, the protein DGC‐2902 harboring a GGDEF diguanylate cyclase moiety was heterologously expressed in *Escherichia coli* together with LOV‐1477. Blue light stimulated cyclic di‐GMP production, but not when the amino acids that act as key phosphorylation sites in the histidine kinase and the REC‐GGDEF protein were absent. Given that the stimulation of apparent enzymatic activity of the thiosulfate dehydrogenase TsdA was not mediated by direct binding of cyclic di‐GMP, 10 PilZ domain proteins, well‐known as receptors for this second messenger, were probed for interactions with TsdA. Two‐hybrid assays homed in on the 123 amino acid long mPilZ‐1753, which interacted with the thiosulfate dehydrogenase TsdA. In line with this finding, the overexpression of the cyclic di‐GMP‐binding mPilZ‐1753 protein stimulated thiosulfate consumption. The precise mechanism of direct binding and activation of catalytic activity of the c‐type cytochrome TsdA remains to be unraveled.

These results are important in several ways. Although cyclic di‐GMP is known to promote the use of carbon and energy sources (He *et al*, 2011), regulate biofilm formation by ocean ecosystem‐relevant electron acceptors, such as nitrate and the biotic sulfur compound dimethylsulfoxide (Martin‐Rodriguez *et al*, 2021), upregulate c‐type cytochromes for ferric ion reduction (Ng *et al*, 2020), promote biofilm formation on energetic minerals for bioleaching (Ruiz *et al*, 2012) and manganese oxidation (Piazza *et al*, 2022), the positive interference with global sulfur cycling and energy conservation had not been demonstrated. As those second messenger interactions can be genus or even species‐specific, it remains to be shown whether cyclic di‐GMP generally promotes thiosulfate oxidation or even interferes with other biogeochemically relevant sulfur cycling pathways. Uncoupling of second messenger reception by mPilZ‐1753 from the apparent activation of enzymatic activity of the thiosulfate dehydrogenase TsdA speaks for a flexible modular system. It also stands to be investigated whether the downstream secretion of sulfur is associated with biofilm formation, which could serve to restrict the localization of the valuable energy source. Finally, exactly how the control of sulfur metabolism by blue light grants an advantage to *Q. flava* warrants further investigation.
